# Translation, cross-cultural adaptation and psychometric evaluation of the Brazilian version of the Cystic Fibrosis Knowledge Scale (CFKS)

**DOI:** 10.1371/journal.pone.0259232

**Published:** 2021-11-16

**Authors:** Karolinne Souza Monteiro, Thayla Amorim Santino, Smita Pakhale, Louise Balfour, Karla Morganna Pereira Pinto de Mendonça

**Affiliations:** 1 Faculty of Health Science of Trairi, Federal University of Rio Grande do Norte, Santa Cruz, Brazil; 2 Graduate Program of Physical Therapy, Federal University of Rio Grande do Norte, Natal, Brazil; 3 Faculty of Medicine, University of Ottawa, Ottawa, Canadá; Monash University, AUSTRALIA

## Abstract

**Background:**

Information on the level of knowledge about cystic fibrosis (CF) among affected people and their families is still scarce.

**Objective:**

This study aimed to translate, cross-culturally adapt and analyze the psychometric properties of the Brazilian version of Cystic Fibrosis Knowledge Scale (CFKS).

**Materials and methods:**

The translation and cross-cultural adaptation involved the stages of translation, synthesis of translations, reverse translation, synthesis of reverse translations, review by a multi-professional committee of experts and pre-testing. The reliability, viability, construct, predictive, concurrent and discriminant validity were investigated.

**Results:**

The sample consisted of 40 individuals with cystic CF, 47 individuals with asthma, 242 healthcare workers and 81 students from the health area. The Brazilian version of the CFKS presented high internal consistency (α = 0.91), moderate floor and ceiling effects, without differences in the test-retest scores. An analysis of factorial exploration identified three dimensions. Confirmatory factor analysis led to an acceptable data-model fit. There was good predictive validity, with a difference in the scores among all the evaluated groups (p <0.001), as well as good discriminant validity since individuals with asthma had greater knowledge of asthma compared to CF (r = 0.401, p = 0.005; r^2^ = 0.162). However, there was no difference between the diagnosis time and knowledge about CF (r = -0.25, p = 0.11; r^2^ = 0.06), either between treatment adherence and knowledge about CF (r = -0.04, p = 0.77; r^2^ = 0.002).

**Conclusion:**

The Brazilian version of the CFKS indicated that the scale is able to provide valid, reliable and reproducible measures for evaluating the knowledge about CF.

## Introduction

Cystic fibrosis (CF) is an autosomal recessive inheritance disease with multisystem involvement and considerable clinical, economic and social burden [1]. Guidelines for its treatment include continuous use of multiple medications, nutritional intervention, respiratory physiotherapy and daily physical activity [2–4], insulin therapy and enzyme replacement in people with pancreatic disorders [3].

Treatment is daily, time consuming, complex and repetitive, which imposes a heavy burden on affected people and their families [5, 6]. These aspects associated with poor knowledge of the disease decrease treatment adherence [6–8]. In turn, low adherence is an important predictor for pulmonary exacerbation [8] and is related to increased risk of hospitalization [9] and longer hospital stay [8].

There has been evidence since the 1990s that inadequate knowledge of CF characterized by a lack of clear understanding of the disease, the benefits of treatment and self-managed care measures, decrease treatment adherence [10]. Current research reinforces this by identifying that people with chronic diseases have little knowledge about medication use, and this is one of the main barriers to treatment adherence [11–13].

Information on the level of knowledge about CF among affected people and their families is still scarce [14]. The knowledge level about the pathology is measured through specific instruments developed for this purpose. There are few validated instruments for the Portuguese language which assess the knowledge of other chronic lung diseases, such as the Asthma Knowledge Questionnaire [15] and the Chronic Obstructive Pulmonary Disease (COPD) Knowledge Questionnaire in Primary Care [16]. There are some instruments regarding CF developed and validated in the English language such as the Cystic Fibrosis Medication Knowledge Questionnaire (CFMKQ) for children and their caregivers [17], a questionnaire assessing the knowledge and understanding of adults with CF [18], the Cystic Fibrosis Knowledge Questionnaire (CFKQ) [19], and the Cystic Fibrosis Knowledge Scale (CFKS) [20], developed for adults. However, there is not yet an instrument available in the Portuguese language to evaluate this construct.

Among these instruments, the CFKS stands out for being a short, self-applied, clinically useful scale that was originally developed and validated in Canada for the English language. It was developed by a panel of 10 CF specialists and a focus group of patients living with CF and their caregivers who elaborated 30 items on the pathology knowledge. There are three answer options for each item: true, false, or not sure. The scale should be interpreted broadly by dividing the number of items answered correctly by the total number of items. The higher the score, the greater the CF knowledge [20]. A previous study aimed to determine the correlation between patients’ confidence in their knowledge and the level of knowledge by CF specific scales, including the CFKS. Participants who received educational content related to CF have performed well on the knowledge instruments. However, patient confidence did not correlate to better knowledge. This study highlighted those educational components should be tailored to patients’ needs and goals. Also, the use of knowledge instruments may be a proper way to identify the gaps [21].

Considering the lack of a valid and reliable instrument for Brazilian adults with CF to assess knowledge about the disease, the aim of this study was to translate, cross-culturally adapt and evaluate the psychometric properties of the Brazilian version of CFKS.

## Materials and method

This study was previously approved by the Research Ethics Committee of the Federal University of Rio Grande do Norte (2,628,776). All participants signed a free and informed consent form.

The translation and cross-cultural adaptation process was previously authorized by the author of the original questionnaire [20] and followed the internationally proposed recommendations [22–24]. In the first stage, two Brazilian translators fluent in English and with different backgrounds, translated the original version of CFKS. Then a committee with three physical therapists (which had experience with translation and cultural adaptation of standardized instruments) compared the two translations and produce one common translated document. In the third stage, two professional translators back translated independently the unified Brazilian version to English. They were native English speakers and had different backgrounds. The committee met to discuss and to consolidate all versions and create a pre-final version of the translated document, which means the first adapted version of CFKS in Brazilian Portuguese.

The multiprofessional committee of experts, which consisted of eight participants, assessed the pre-final Brazilian version of CFKS. Subsequently, the pretest was performed with 10 subjects with CF and 10 health professionals using the pre-final version. Each volunteer answered an online version of CFKS. When evaluating each item, the volunteer could select either “it is clear/understood” or “it is not clear/not understood”. The volunteer could suggest modifications to make it understandable [25]. To the final stage of the adaptation process, all the versions were submitted and approval by original author.

### Participants

Similar to the validation of the original CFKS version [20], the population was composed of adults diagnosed with CF according to the Brazilian Guidelines for the Diagnosis and Treatment of Cystic Fibrosis [3]; Health professionals; adults diagnosed with asthma according to the Global Initiative for Asthma (GINA) [26]; and undergraduate health students (physiotherapy). All participants were ≥18 years old. Participants who could not understand any of the necessary procedures and/or did not answer the questionnaires during the pre-defined period would be excluded. Those among participants with CF who underwent lung transplantation would be excluded.

The recruitment of health professionals, CF and asthma participants was carried out through social media dissemination and in a reference hospital in the treatment of respiratory diseases. Physiotherapy students were recruited from a university.

Contact was made through e-mail disclosure to regional and national councils of health professionals, CF associations and non-governmental organizations, social networks and on a previously scheduled date, in face-to-face situations. After signing the consent form and agreeing to participate in the research, the procedures for data collection were carried out.

### Data collection

**Fig 1:**
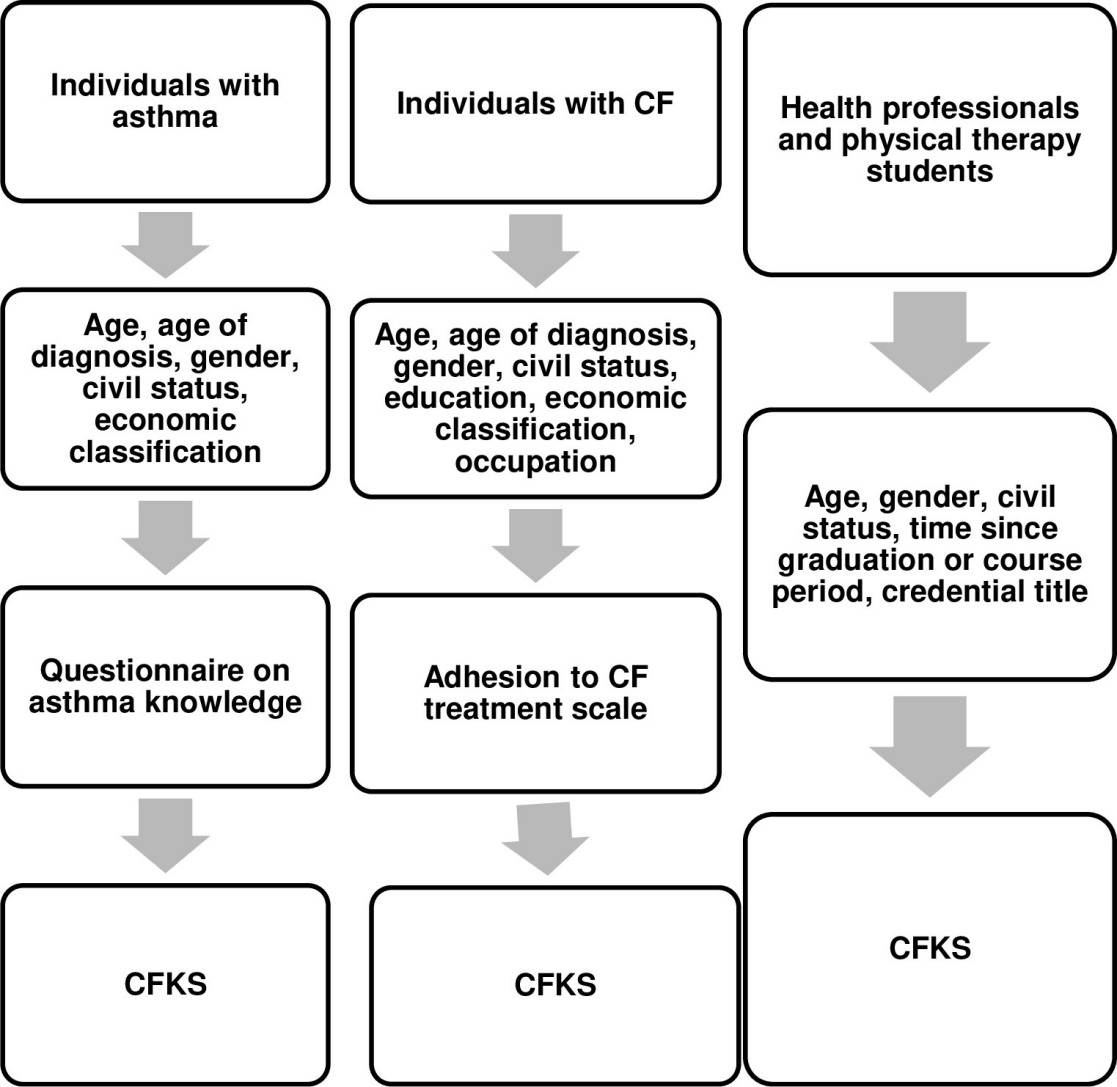
Flowchart of the evaluations in different samples.

Prior to applying the Brazilian version of CFKS, participants answered an economic classification questionnaire [27], a questionnaire to assess CF patient adherence to treatment [28] and a scale on asthma knowledge [15] (Fig 1).

The economic classification was performed according to the criteria defined by the Brazil Economic Classification Criterion (CCEB). CCEB is based on a point system that considers the number of durable goods, level of education, and access to public services. Higher points indicate higher economic class: A, from 45 to 100 points; B1, from 38 to 44 points; B2, from 29 to 37 points; C1, from 23 to 28 points; C2, from 17 to 22 points; D and E, from zero to 16 points [27].

Adherence to treatment by the CF participants was assessed by a questionnaire developed by Conway et al. (1996) [29] and adapted to the Portuguese language [28]. However, it has not been validated to Brazilian population and there is a lack of validated instruments measuring this construct. The adhesion score considers the quotient between the total points obtained and the total possible points, ranging from 1 (total adhesion) to 0 (zero adhesion).

The asthma knowledge questionnaire has 34 items assessing etiology, pathophysiology, symptoms, triggers, treatment, prevention, and action plans. Items can be scored as “yes”, “no” and “I don’t know” with a maximum score of 34 points. The Brazilian version demonstrated a unidimensional structure through factor analysis (Kaiser-Meyer-Olkin = 0.53; Bartlett’s test = p < 0.001), good test-retest reliability (p = 0.43), and adequate internal consistency (α = 0.69) [15].

### Statistical analysis

Data were analyzed according COnsensus-based Standards for the selection of health Measurement INstruments (COSMIN) [23]. The analyses were performed using the Statistical Package for Social Science (SPSS) software, version 22.0 for Windows (IBM Corporation, Armonk, NY, USA), considering a significance level of 5%. Data normality was verified by the Kolmogorov-Sminorv test.

The effect size (ES) of correlations were computed using the online calculator https://www.psychometrica.de/correlation.html, by r^2^ [30]. According to Cohen, the reference values are: low effect: < .12; medium effect: .13 to .25; high effect: ≥ .26 or above [31]. The ES of comparison analysis was calculated by Cohen’s d in the online calculator https://www.psychometrica.de/effect_size.html [32]. Cohen’s was interpreted according to the guidelines suggested by Cohen (small effect: .20 to .49; moderate effect: .50 to .79; and ≥ .80, strong effect) [33].

#### Content validity

Content validity was analyzed by the Content Validation Index (CVI), accepting values ≥0.75 during the multiprofessional expert committee steps [34].

#### Reliability

Internal consistency was analyzed using Cronbach’s alpha coefficient. Values of α above 0.70 were considered adequate, confirming that the items were sufficiently correlated [35].

#### Test-retest

The Brazilian version of CFKS was applied to undergraduate students (Physiotherapy) who had not taken courses on studying CF. The test-retest was performed every two weeks. The hypothesis was that there would be no differences between the test and the retest, since the students would not have activities related to CF knowledge. The evaluation was performed by the Intraclass Correlation Coefficient (ICC), for which 0.70 is recommended as a minimum standard for reliability [36].

#### Floor and ceiling effects

The ceiling effect was determined by the percentage of participants who scored in the upper decile of the scale, and the floor effect by the percentage of participants who scored in the lower decile of the scale. Floor and ceiling effects are considered to be present if more than 15% of participants have minimal or maximal scores [37]. Moderate floor and ceiling effects were considered up to 25%, and substantial if greater than 25% [38].

#### Construct validity

The construct validity of the instrument was assessed by exploratory (EFA) and confirmatory factor analysis (CFA). The Kaiser-Meyer-Olkin (KMO) test, Barlett Sphericity test and EFA were performed. Oblique processes was used for matrix rotation to obtain the best factor load configuration and factor interpretability. A CFA was performed with JASP software (https://jasp-stats.org/). The fit indices were calculated by χ^2^, degree of freedom (df), χ^2^/df, comparative fit index (CFI), Tucker-Lewis Index (TLI), goodness of fit index (GFI), standardized root mean residual (SRMR), and root-mean-square error of approximation (RMSEA). The reference values for excellent model fit were χ^2^/df < 3, CFI, TLI, and GFI values > 0.95, SRMR < 0.08, and RMSEA ≤ 0.06 [23, 39]. Estimation method was performed using robust Diagonally Weight Listed Square (DWLS) [40].

#### Criterion validity

Criterion validity considers the relationship between scores of a given instrument and some external criterion. The scores of the measuring instrument must be correlated with the scores of the external criterion and this coefficient is analyzed. Criterion validity could be analyzed by predictive and concurrent validity [23].

#### Predictive validity

Predictive validity was performed by comparing the percentage of correct answers from the four groups: participants with CF, participants with asthma, health professionals and physical therapy students, using the Kruskal-Wallis test.

#### Concurrent validity

Pearson’s correlation test was used to correlate CF diagnosis time and treatment adherence with CF knowledge. The values recommended by the British Medical Journal were considered as reference [41]. An unpaired student’s t-test was used to compare patients’ gender and knowledge about CF.

#### Discriminating validity

Discriminating validity assessed whether the CFKS was unduly related to different constructs [42]. This validity was assessed in participants with asthma, correlating their knowledge of CF to their knowledge of asthma by the Pearson’s correlation test. The hypothesis was that there was a positive correlation between the responses of both instruments.

## Results

### Sample characteristics

The sample consisted of 411 participants, 40 (9.7%) participants with CF, 242 (58.9%) health professionals, 82 (20%) physical therapy students and 47 (11.4%) participants with asthma. The socioeconomic characteristics of the evaluated sample are described in Table 1.

**Table 1:** Socioeconomic characterization of cystic fibrosis participants, health professionals, physiotherapy students and participants with asthma.

Variable	M±SD or N (%)
**Participants with CF (n = 40)**	
Age, in years	23.85±7.27
Age of diagnosis, in years	9.45±8.70
Gender	
Female	29 (72.50)
Male	11 (27.50)
Civil status	
Single/Widowed/Divorced	26 (65.00)
Married/Stable union	14 (35.00)
Education	
Incomplete elementary	2 (5.00)
Completed elementary	1 (2.50)
Incomplete High school	5 (12.50)
Completed High school	10 (25.00)
Incomplete Higher education	8 (20.00)
Completed Higher education	6 (15.00)
Post-graduation	8 (20.00)
Economic classification	
A	1 (2.50)
B	22 (55.00)
C	12 (30.00)
D-E	5 (12.50)
Current occupation	
Employed	8 (20.0)
Unemployed	22 (55.00)
Pension/Retired	10 (25.00)
Adhesion to cystic fibrosis treatment	0.73 (0.20)
**Health professionals (n = 242)**	
Age, in years	30.98±7.13
Time since graduating, in years	6.79 (6.74)
Gender	
Female	202 (83.5)
Male	40 (16.5)
Credential title	
Doctorate degree	13 (5.40)
Master’s degree	54 (22.30)
Specialization	103 (42.60)
Graduate degree	72 (29.70)
**Physiotherapy students (n = 82)**	
Age, in years	20.99±3.86
Gender	
Female	62 (75.60)
Male	20 (24.4)
**Participants with asthma (n = 47)**	
Age, in years	29.96±9.16
Age of diagnosis, in years	9.60±8.56
Gender	
Female	36 (76.60)
Male	11 (23.40)
Civil status	
Single/Widowed/Divorced	28 (59.60)
Married/Stable union	19 (40.40)
Economic classification	
A	7 (14.90)
B	20 (42.60)
C	18 (38.20)
D-E	2 (4.30)

### Content validity

The multi-professional committee of experts was composed of eight specialists with an average age of 49.75 ± 9.11 years, six (75.00%) females and five (62.50%) with doctorate degrees. The committee members were two physiotherapists, three physicians, a nutritionist, a psychologist with experience in psychometrics and a CF patient’s mother, representing the target audience.

Two items of the Brazilian version of CFKS were considered inappropriate (CVI < 0.75): a) item 4: “Aerobic exercises are more important for CF patients than for patients without CF” and b) item 5: “For greater social support, face-to-face interaction among CF patients is highly recommended” with CVI of 0.63 and 0.38, respectively. From the suggestions of the multidisciplinary committee of experts, these items were adjusted to: a) item 4: “Aerobic exercise (e.g., running, swimming, cycling) is more important for CF patients than for patients without CF” and b) item 5: “For better socialization, face-to-face interaction between CF patients is recommended”. These items reached CVI of 1.00 and 0.80 after the committee’s suggestions, respectively.

The pretest was performed with 10 female health professionals with a mean age of 35.40 ± 9.70 years; and with 10 CF patients, being 60% women, with a mean age of 23.30 ± 5.98 years. All items on the scale obtained CVI ≥ 0.75 on the pretest and therefore did not require further modifications.

The Brazilian version of CFKS is on S1 Appendix.

### Psychometric properties of the Brazilian version of CFKS

#### Reliability

The Brazilian version of CFKS presented high internal consistency of its items, identified by Cronbach’s alpha coefficient (0.91). Detailed information is shown in Table 2.

**Table 2:** Mean scores from the Brazilian version of the Cystic Fibrosis Knowledge Scale (CFKS), standard deviation (SD) for individual items, correlated item-corrected total, and internal consistency (Cronbach’s alpha) if item is excluded.

Scale item	Mean	SD	Item correlation/corrected total	Cronbach’s alpha if item is excluded
1. Type of diet	0.95	0.85	0.35	0.91
2. Need to do chest physiotherapy	0.43	0.79	0.59	0.91
3. Proper nutrition and progression of lung disease	0.32	0.73	0.54	0.91
4. Importance of aerobic exercise	0.81	0.82	0.44	0.91
5. Recommendation of face-to-face socialization	0.65	0.79	0.31	0.91
6. Lung transplants	0.81	0.91	0.56	0.91
7. Risk of developing diabetes	1.14	0.93	0.53	0.91
8. Having a child with CF	1.25	0.53	0.48	0.91
9. Use of inhaled CF medications	0.50	0.80	0.56	0.91
10. Use of antibiotics and drug resistance	0.33	0.70	0.49	0.91
11. Medications for drying up mucus	0.49	0.81	0.54	0.91
12. Assistance during chest physiotherapy	1.18	0.62	0.49	0.91
13. Use of masks to deliver nebulized medication	1.27	0.77	0.34	0.91
14. Frequency of cleaning nebulizer devices per month	0.96	0.62	0.40	0.91
15. Body position during chest physiotherapy	1.14	0.72	0.40	0.91
16. Calories eaten	1.24	0.68	0.49	0.91
17. When to use antibiotics	0.90	0.76	0.46	0.91
18. Use of soluble vitamins	0.90	0.97	0.54	0.91
19. Frequency of cleaning nebulizer devices per week	0.51	0.75	0.48	0.91
20. Consume of pancreatic enzyme supplements	1.21	0.90	0.53	0.91
21. Genetic screening test	0.74	0.95	0.57	0.91
22. Vitamin deficiency	0.63	0.92	0.67	0.90
23. Pancreatic enzymes and risk of drug resistance	1.09	0.92	0.29	0.91
24. Male infertility	1.24	0.90	0.52	0.91
25. Routine of chest physiotherapy	1.09	0.38	0.48	0.91
26. Benefits of chest physiotherapy	1.09	0.45	0.48	0.91
27. Lung functioning and body weight	0.77	0.90	0.49	0.91
28. CF genes	0.97	0.87	0.57	0.91
29. Amount of pancreatic enzyme supplements	1.28	0.89	0.52	0.91
30. Safety of face-to-face socialization	1.31	0.67	0.46	0.91

#### Test-retest

Test-retest was evaluated in a sample of 57 students. There was no difference in the 2-week interval (ICC: 0.813; 95% CI 0.682–0.890; p<0.001), suggesting adequate test-retest reliability.

#### Floor and ceiling effects

For the total of CFKS score no floor or ceiling effects were found as floor effect reached 7.3%, while the ceiling effect was found in only 4.9% of the total sample.

#### Construct validity

The results of the Kaiser-Meyer-Olkin test (KMO = 0.93) and Barlett’s sphericity test (χ^2^ = 3737.54; p = 0.00) enabled performing an exploratory factor analysis (Table 2). Component extraction was performed based on the determination of three factors, which explained 40.06% of the total variance (Table 3).

**Table 3:** Factorial structure of the Brazilian version of the Cystic Fibrosis Knowledge Scale (CFKS).

Scale Item	Factors			
	1	2	3	h^2^
19. Frequency of cleaning nebulizer devices per week	0.67			0.43
14. Frequency of cleaning nebulizer devices per month	0.66			0.36
15. Body position during chest physiotherapy	0.60			0.32
9. Use of inhaled CF medications	0.58			0.43
17. When to use antibiotics	0.56			0.35
26. Benefits of chest physiotherapy	0.56			0.41
12. Assistance during chest physiotherapy	0.52			0.35
10. Use of antibiotics and drug resistance	0.52			0.33
27. Lung functioning and body weight	0.49			0.32
25. Routine of chest physiotherapy	0.47			0.36
2. Need to do chest physiotherapy	0.47			0.42
6. Lung transplants	0.45			0.46
11. Medications for drying up mucus	0.42			0.36
13. Use of masks to deliver nebulized medication	0.41			0.18
3. Proper nutrition and progression of lung disease	0.38			0.38
5. Recommendation of face-to-face socialization	0.37			0.34
4. Importance of aerobic exercise	0.26			0.24
30. Safety of face-to-face socialization	0.23			0.27
29. Amount of pancreatic enzyme supplements		0.79		0.57
20. Consume of pancreatic enzyme supplements		0.78		0.57
7. Risk of developing diabetes		0.75		0.54
24. Male infertility		0.70		0.49
28. CF genes		0.58		0.45
18. Use of soluble vitamins		0.56		0.41
21. Genetic screening test		0.52		0.42
22. Vitamin deficiency		0.51		0.54
8. Having a child with CF		0.33		0.34
1. Type of diet			0.73	0.57
23. Pancreatic enzymes and risk of drug resistance			0.60	0.39
16. Calories eaten			0.46	0.43
**Eigenvalue**	8.73	2.00	1.29	
**Percentage of explained variance**	29.11	6.66	4.29	
**Cronbach’s alpha**	0.86	0.86	0.52	

h^2^: communalities. Notes: a) Extraction method: principal component analysis. B) Rotation Method: Direct Oblimin with Kaiser Normalization.

The factorial analysis allowed us to identify the three dimensions that make up the Brazilian version of CFKS, namely: 1) “medication, physiotherapeutic aspects and social interaction”, collecting 18 items related to knowledge about the use of inhaled medications and hygiene of the equipment used to administer them; antibiotic use, respiratory physiotherapy and face-to-face interaction among people with CF; 2) “gastrointestinal, genetic and reproductive aspects”, collecting 9 items related to vitamin and enzymatic supplementation, diabetes, genetics and fertility; and 3) “nutritional aspects”, which brought together 3 items with specific information on dietary lipid and caloric content and inadequate administration of pancreatic enzymes (Table 3).

After EFA, the CFA was performed with a three-dimensional model. The indexes of x^2^, df, χ^2^/df, CFI, TLI, GFI, SRMR, and RMSEA values indicate an excellent fit to the data (x^2^ = 389.597, df = 402, p = 0.662, χ^2^/df = 0.969, CFI = 1.000, TLI = 1.001, GFI = 0.988, SRMR = 0.056, RMSEA = 0.000, Confidence interval = 0.000–0.019).

#### Predictive validity

The Kruskal-Wallis test showed that there is a difference between the groups regarding the percentage of correct answers (x^2^(3) = 192.757; p<0.001). Differences occurred between groups of physiotherapy students and health professionals, physiotherapy students and CF participants, asthma participants and health professionals, asthma participants and CF participants, health professionals and CF participants (p<0.001 for all comparisons) (S2 Appendix).

#### Concurrent validity

The CF patients had a high percentage of knowledge about the disease (79.50 ± 10.22), however there was no relationship between the time since diagnosis and the percentage of correct answers (r = -0.25; p = 0.11; r^2^ = 0.06), there was no relationship between treatment adherence and knowledge (r = -0.04; p = 0.77; r^2^ = 0.002), and there was no difference between genders (p = 0.40; 95% CI: -4.28; 10.42; Cohen’s d = -0.29).

#### Discriminating validity

There was a moderate and positive correlation between CF knowledge (33.40 ± 25.40) and asthma (24.77 ± 4.54) (r = 0.401; p = 0.005; r^2^ = 0.162).

## Discussion

The translation, cross-cultural adaptation and evaluation of the psychometric properties of the Brazilian version of CFKS indicated that the scale is capable of providing reliable, valid and reproducible measurements in evaluating the construct knowledge about CF. The method used in this study has been internationally used and accepted to ensure methodological quality [22–24].

Beaton et al. (2000) [22] recommend that the translators involved in the process should have different profiles, one of which should have technical knowledge and be aware of the concepts to be evaluated, while the other should represent the population of the country to which the instrument is being translated. The multi-professional committee of experts should involve health professionals, a specialist in the translation and cross-cultural adaptation process, and a representative of the target population. All these recommendations were followed, which ensured the conceptual, semantic and idiomatic equivalence of this version. In addition, the CVI ≥ 0.75 ensured that items fit the new cultural context. In developing the original English scale [20], the authors attested to its content validity only through a panel of experts.

Regarding reliability, both the original version [20] and the Brazilian version of CFKS were reliable (α>0.90). This was different from the reliability presented by the instrument of Siklosi et al. (2010) [18] (α = 0.60), confirming that the items were not sufficiently correlated [35]. In addition, the instrument proposed by Nash et al. (2009) [19] is only available in summary form and did not enable a comparison of the reliability of this scale with the others available [18, 20].

Similar to the original version [20], the test-retest was performed with students instead of CF patients and showed no differences when applied at two-week intervals. The floor and ceiling effects were moderate, showing the ability of the instrument to detect high and low levels of CF knowledge, being similar to its original version.

Due to the multidimensional nature of the construct, the validation study of the original CFKS version performed an exploratory factor analysis, only identifying one factor which explained 42.2% of the total variance. In the Brazilian CFKS version, 40.06% of the total variance was explained by three factors, according to the nature of the grouped items. Thus, the Brazilian version of CFKS has four scores; one total score and the individual scores for each of the extracted dimensions.

The calculation of the total scale score is performed by the number of correctly answered items divided by 30. The dimension score “medication, physical therapy and social interaction” is calculated by the number of correctly answered items divided by 18. The dimension score “gastrointestinal, genetic and reproductive aspects” is performed by the number of correctly answered items divided by nine. Finally, the “nutritional aspects” dimension score is calculated by the number of correct items divided by three.

Currently, only the Brazilian version of CFKS presents a subclassification and theoretical interpretation of the dimensions extracted through EFA and with an acceptable CFA fit model. The identification of different dimensions will enable health professionals to verify which dimension may be the knowledge gap of CF individuals with greater precision. This identification will make it possible to relate knowledge gaps about the disease with health outcomes such as treatment adherence, disease exacerbation and quality of life. Moreover, educational interventions may be directed to the needs of each individual in order to obtain satisfactory results in the clinical control of CF patients. Current studies have highlighted the importance of knowledge about the disease to increase treatment adherence [11–13, 43], and thereby reduce the number of hospitalizations and increase the life expectancy of people with CF [3, 8, 9, 44].

As hypothesized, the samples in this study presented different knowledge levels about CF, confirming the predictive validity of the Brazilian version of CFKS [45]. Health professionals in the original study obtained higher scores, followed by participants with CF. However, the authors did not describe which categories of health professionals responded to the instrument. The lack of this information prevented a more accurate comparative analysis on the knowledge level of health professionals in both cultural contexts.

The items with the lowest hit percentage in the group of participants with CF were related to the use of nebulized medications, positioning during respiratory physiotherapy and drug resistance. Meanwhile, the lowest hit rate in the group of professionals was observed in the items related to treatment, genetics and social interaction. These aspects raise the need for adequacy in training health professionals in Brazil in order to deepen the knowledge about CF, and from this be able to offer increasingly early diagnosis and appropriate treatments. In addition, these professionals need to be able to deliver quality educational interventions to patients and their families [46].

Regarding the concurrent validity, participants with CF who had greater adherence to treatment obtained higher scores on knowledge of the disease in the original CFKS version [20]. On the other hand, our study did not identify this relationship. It is necessary to interpret this data with caution, since the scale used in our study to assess treatment adherence was an invalidated instrument, adapted by Dalcin et al. (2009) [28]. Thus, the instrument may not have adequate psychometric properties to assess adherence construct. There are no scales available in Brazil to assess patient adherence to CF treatment. We believe that this gap should not exist, considering the coexistence between knowledge, adherence and efficacy in treating CF patients [11, 12].

Regarding the discriminant validity assessment, the findings of the present study support those observed in the validation study of the original CFKS version [20], as both studies showed a significant correlation between the asthma knowledge scale and the CFKS. In addition to participants with asthma, the original study compared the knowledge of participants with COPD using a COPD knowledge scale and CFKS. Participants with COPD were not evaluated in the present study, as there is no Portuguese validated instrument to assess this construct.

This study evaluated a large population of CF participants from the Northeast, Midwest, South and Southeast of Brazil. These patients represented different economic classes, marital statuses and education levels. However, the study was limited to applying questionnaires through an online platform, which may have limited the participation of patients without internet access. The lack of treatment adherence scales for CF participants and COPD knowledge translated and validated in Portuguese was limited, but did not prevent reproducing the psychometric property evaluation process to be similar to the original study.

Although the CFKS was developed for CF participants, it can be used to assess and promote the knowledge of health professionals in Brazil. Even with the neonatal screening test implemented in all countries, which evaluates the presence of CF, patients in our study were diagnosed with CF at an average age of nine years old. This data draws attention when compared with the average time of diagnosis observed in Europe and North America, which are 3.6 months and 3 years, respectively [47, 48]. Health professionals’ lack of knowledge about the disease contributes to a late CF diagnosis, compromising patients’ survival.

In conclusion, the translation process, cross-cultural adaptation and evaluation of psychometric properties indicated that the Brazilian CKFS version is able to provide valid and reliable measures for evaluating CF knowledge in the current cultural context. Therefore, this study has relevance to clinical practice and scientific research, and may help in both training health professionals and to improve self-knowledge of CF participants. In general, the Brazilian CFKS will enable knowledge screening in terms of crucial aspects for disease management that are lacking. Also, this instrument may be an important tool in decision-making processes, especially during educational interventions and when planning or implementing public policies for this population.

## Supporting information

S1 Appendix Brazilian version of CFKS. (DOCX)

S2 Appendix Absolute and relative frequency of correct answers from the Brazilian version of the Cystic Fibrosis Knowledge Scale (CFKS) by group, total, and scale dimension. (DOCX)

S1 Data (XLSX)
