# Supplementary material for: Translation, cross-cultural adaptation and psychometric evaluation of the Brazilian version of the Cystic Fibrosis Knowledge Scale (CFKS)
Source: PLoS One. 2021 Nov 16;16(11):e0259232. doi: 10.1371/journal.pone.0259232 (PMC8594816; doi:10.1371/journal.pone.0259232)
Supplement: S1 Appendix — (DOCX) [file pone.0259232.s001.docx]

**S1 Appendix: ESCALA DE CONHECIMENTO SOBRE A FIBROSE CÍSTICA (FC)**

**Instruções: Por favor, marque Verdadeiro, Falso ou Não Sei para cada item.**

|  | **Verdadeiro** | **Falso** | **Não sei** |
| --- | --- | --- | --- |
| 1. Pacientes com FC necessitam ter uma dieta com pouca gordura. |  |  |  |
| 2. Pacientes com FC devem fazer fisioterapia respiratória pelo menos uma vez por dia. |  |  |  |
| 3. A doença pulmonar em pacientes com FC pode piorar com uma nutrição inadequada |  |  |  |
| 4. Exercícios aeróbicos (ex: correr, nadar, andar de bicicleta) são mais importantes para pacientes com FC do que para pacientes sem FC. |  |  |  |
| 5. Para uma melhor socialização, a interação presencial entre pacientes com FC é recomendada. |  |  |  |
| 6. O transplante de pulmão é a última opção de tratamento para alguns pacientes com FC. |  |  |  |
| 7. Pacientes com FC têm maior risco de desenvolver diabetes do que pacientes sem FC. |  |  |  |
| 8. Todas as mulheres com FC que engravidarem terão filhos com FC. |  |  |  |
| 9.  Medicamentos inalatórios (ex: bombinha, nebulização) para FC ajudam a soltar e eliminar as secreções do pulmão. |  |  |  |
| 10. Não tomar os antibióticos como prescritos pode levar ao desenvolvimento de resistência ao medicamento (as bactérias deixam de ser destruídas). |  |  |  |
| 11. Medicamentos para eliminar a secreção são úteis para pacientes com FC. |  |  |  |
| 12. A fisioterapia respiratória em pacientes com FC somente pode ser realizada com a assistência de outra pessoa. |  |  |  |
| 13. Máscaras usadas para administrar a medicação por nebulização devem ser mantidas de 3 à 5 cm do rosto. |  |  |  |
| 14. Os equipamentos usados para administrar medicações inalatórias precisam ser limpos mensalmente. |  |  |  |
| 15. Durante a fisioterapia respiratória, pacientes com FC devem permanecer em uma posição fixa para melhorar a eliminação da secreção. |  |  |  |
| 16. Pacientes com FC precisam ingerir menos calorias do que pacientes sem FC. |  |  |  |
| 17. Pacientes com FC precisam usar antibióticos para tratar infecções virais. |  |  |  |
| 18. Pacientes com FC devem ingerir vitaminas lipossolúveis (ou seja, A, D, E, K) diariamente. |  |  |  |
| 19. Os equipamentos usados para administrar medicações inalatórias precisam ser limpos pelo menos uma vez por semana. |  |  |  |
| 20. Pacientes com FC devem tomar suplementos de enzimas pancreáticas em todas as refeições e lanches. |  |  |  |
| 21. Um teste genético está disponível para identificar FC. |  |  |  |
| 22. Pacientes com FC têm risco de desenvolver deficiência de vitaminas. |  |  |  |
| 23. Tomar enzimas pancreáticas de forma inadequada pode levar ao desenvolvimento de resistência a medicamentos. |  |  |  |
| 24. A maioria dos homens com FC é infértil. |  |  |  |
| 25. Não é necessário fazer fisioterapia respiratória nos dias em que os pacientes com FC se sentem bem. |  |  |  |
| 26. As técnicas de fisioterapia respiratória são benéficas apenas quando os pacientes com FC desenvolvem sintomas. |  |  |  |
| 27. O funcionamento dos pulmões está diretamente relacionado ao peso corporal saudável em pacientes com FC. |  |  |  |
| 28. Ambos os pais devem ter o gene da FC para que uma criança nasça com a doença. |  |  |  |
| 29. Se você tomar menos do que a quantidade correta de suplementos de enzimas pancreáticas, você evacuará com mais frequência. |  |  |  |
| 30. A interação presencial entre dois pacientes com FC com a mesma bactéria (e.g. Pseudomonas aeruginosa (PA)/Cepacia) é segura. |  |  |  |
| Obrigada por responder a este questionário |  |  |  |

**CYSTIC FIBROSIS (CF) KNOWLEDGE SCALE**

**Instructions: Please check True, False or Don’t Know for each item.**

|  | **True** | **False** | **Don’t know** |
| --- | --- | --- | --- |
| 1. CF patients need to eat a low fat diet. |  |  |  |
| 2. CF patients need to do chest physiotherapy (CPT) at least once per day. |  |  |  |
| 3. Proper nutrition can delay the progression of lung disease in CF patients. |  |  |  |
| 4. Aerobic exercise is more important for CF patients than non-CF patients. |  |  |  |
| 5. For increasing social support, face-to-face socializing between CF patients is highly recommended. |  |  |  |
| 6. Lung Transplants are a last resort option for some CF patients |  |  |  |
| 7. CF patients are at greater risk of developing diabetes than those without CF. |  |  |  |
| 8. All women with CF who get pregnant will have children with CF disease. |  |  |  |
| 9. Inhaled CF medications help to loosen lung secretions in order to promote mucus clearance. |  |  |  |
| 10. Not taking antibiotics as prescribed can lead to the development of drug resistance. |  |  |  |
| 11. Medications for drying up mucus are useful for CF patients. |  |  |  |
| 12. Chest physical therapy in CF patients can only be done with the assistance of another person. |  |  |  |
| 13. Masks used to deliver nebulized medication should be kept 3-5 cm away from the face. |  |  |  |
| 14. Delivery devices used for inhaled medication need to be cleaned monthly. |  |  |  |
| 15. During chest physiotherapy (CPT), CF patients should remain in one fixed position to improve mucus clearance. |  |  |  |
| 16. CF patients need to eat less calories than those without CF. |  |  |  |
| 17. CF patients need to use antibiotics to treat viral infections. |  |  |  |
| 18. CF patients should take fat soluble Vitamins (i.e. A, D, E, K) daily. |  |  |  |
| 19. Delivery devices used for inhaled medication need to be cleaned at least once per week. |  |  |  |
| 20. CF patients should take pancreatic enzyme supplements with all meals and snacks. |  |  |  |
| 21. A genetic screening test is available for CF. |  |  |  |
| 22. CF patients are at risk of developing a vitamin deficiency. |  |  |  |
| 23. Taking pancreatic enzymes improperly can lead to the development of drug resistance. |  |  |  |
| 24. Most males with CF are infertile. |  |  |  |
| 25. Doing chest physiotherapy is not necessary on days when CF patients feel well. |  |  |  |
| 26. Chest physiotherapy techniques are only beneficial once CF patients develop symptoms. |  |  |  |
| 27. Lung functioning is closely related to healthy body weight in CF patients. |  |  |  |
| 28. Both parents must carry the CF gene for a child to be born with CF disease. |  |  |  |
| 29. If you take less than the correct amount of pancreatic enzyme supplements it will result in increased stool frequency. |  |  |  |
| 30. Face-to-face socialization between two CF patients with the same bug (e.g. Pseudomonas aeruginosa (PA)/Cepacia) is safe. |  |  |  |
| Thank you for completing this questionnaire. |  |  |  |

Balfour L, Armstrong M, Holly C, Gaudet E, Aaron S, Tasca G, et al. Development and psychometric validation of a cystic fibrosis knowledge scale. Respirology. 2014;19(8):1209–14.
