# Supplementary material for: Translation, cross-cultural adaptation and psychometric evaluation of the Brazilian version of the Cystic Fibrosis Knowledge Scale (CFKS)
Source: PLoS One. 2021 Nov 16;16(11):e0259232. doi: 10.1371/journal.pone.0259232 (PMC8594816; doi:10.1371/journal.pone.0259232)
Supplement: S2 Appendix — (DOCX) [file pone.0259232.s002.docx]

**S2 Appendix:** Absolute and relative frequency of correct answers from the Brazilian version of the Cystic Fibrosis Knowledge Scale (CFKS) by group, total, and scale dimension.

| **Scale Item** | **Total sample**  **n=411** | **Patients with CF**  **n=40** | **Health professionals**  **n=242** | **Physiotherapy students**  **n=81** | **Patients with asthma**  **n=47** |
| --- | --- | --- | --- | --- | --- |
| 1. Type of diet (F) | 114 (27.7) | 32 (80.0) | 74 (30.6) | 5 (6.2) | 3 (6.4) |
| 2. Need to do chest physiotherapy (T) | 312 (75.9) | 39 (97.5) | 206 (85.1) | 43 (53.1) | 24 (51.1) |
| 3. Proper nutrition and progression of lung disease (T) | 342 (83.2) | 40 (100) | 219 (90.5) | 54 (66.7) | 28 (59.6) |
| 4. Importance of aerobic exercise (T) | 182 (44.3) | 34 (85.0) | 104 (43.0) | 24 (29.6) | 20 (42.6) |
| 5. Recommendation of face-to-face socialization (F) | 116 (28.3) | 30 (75.0) | 76 (31.4) | 7 (8.8) | 3 (6.4) |
| 6. Lung transplants (T) | 214 (52.1) | 32 (80.0) | 142 (58.7) | 22 (27.2) | 17 (36.2) |
| 7. Risk of developing diabetes (T) | 150 (36.5) | 38 (95.0) | 99 (40.9) | 5 (6.2) | 8 (17.0) |
| 8. Having a child with CF (F) | 268 (65.2) | 37 (92.5) | 172 (71.1) | 34 (42.0) | 25 (53.2) |
| 9. Use of inhaled CF medications (T) | 284 (69.1) | 35 (87.5) | 185 (76.4) | 40 (49.4) | 24 (51.1) |
| 10. Use of antibiotics and drug resistance (T) | 330 (80.3) | 38 (95.0) | 208 (86.0) | 53 (65.4) | 30 (63.8) |
| 11. Medications for drying up mucus (T) | 291 (70.8) | 39 (97.5) | 177 (73.1) | 48 (59.3) | 26 (55.3) |
| 12. Assistance during chest physiotherapy (F) | 238 (57.9) | 34 (85.0) | 168 (69.4) | 18 (22.2) | 18 (38.3) |
| 13. Use of masks to deliver nebulized medication (F) | 134 (38.8) | 23 (57.5) | 98 (40.5) | 0 | 13 (27.7) |
| 14. Frequency of cleaning nebulizer devices per month (F) | 251 (61.4) | 24 (60.0) | 183 (75.6) | 28 (34.6) | 16 (34.0) |
| 15. Body position during chest physiotherapy (F) | 188 (45.7) | 21 (52.5) | 145 (59.9) | 7 (8.6) | 15 (31.9) |
| 16. Calories eaten (F) | 196 (47.7) | 39 (97.5) | 141 (58.3) | 6 (7.4) | 10 (21.3) |
| 17. When to use antibiotics (F) | 166 (40.4) | 4 (10.0) | 134 (55.4) | 14 (17.3) | 14 (29.8) |
| 18. Use of soluble vitamins (T) | 215 (52.3) | 35 (87.5) | 141 (58.3) | 22 (27.5) | 17 (36.2) |
| 19. Frequency of cleaning nebulizer devices per week (T) | 263 (64.0) | 30 (75.0) | 163 (67.4) | 44 (54.3) | 26 (55.3) |
| 20. Consume of pancreatic enzyme supplements (T) | 131 (31.9) | 34 (85.0) | 86 (35.5) | 5 (6.2) | 6 (12.8) |
| 21. Genetic screening test (T) | 248 (60.3) | 36 (90.0) | 180 (74.4) | 17 (21.0) | 15 (31.9) |
| 22. Vitamin deficiency (T) | 60 (14.6) | 36 (90.0) | 201 (83.1) | 23 (28.4) | 15 (31.9) |
| 23. Pancreatic enzymes and risk of drug resistance (F) | 60 (14.6) | 14 (35.0) | 44 (18.2) | 2 (2.5) | 0 |
| 24. Male infertility(T) | 125 (30.4) | 33 (82.5) | 86 (35.5) | 1 (1.2) | 5 (10.6) |
| 25. Routine of chest physiotherapy (F) | 348 (84.7) | 40 (100.0) | 214 (88.4) | 67 (82.7) | 27 (57.4) |
| 26. Benefits of chest physiotherapy (F) | 323 (78.6) | 33 (82.5) | 209 (86.4) | 58 (71.6) | 23 (48.9) |
| 27. Lung functioning and body weight (T) | 222 (54.0) | 24 (60.0) | 148 (61.2) | 33 (40.7) | 17 (36.2) |
| 28. CF genes (T) | 158 (38.4) | 37 (92.5) | 104 (43.0) | 9 (11.1) | 8 (17.0) |
| 29. Amount of pancreatic enzyme supplements (T) | 117 (28.5) | 35 (87.5) | 71 (29.3) | 5 (6.2) | 6 (12.8) |
| 30. Safety of face-to-face socialization (F) | 190 (46.2) | 28 (70.0) | 130 (53.7) | 20 (24.7) | 12 (25.5) |
| Mean (SD) | 52.14 (23.55) | 79.50 (10.22) | 59.33 (17.27) | 28.29 (14.10) | 33.40 (25.40) |
| Dimensions |  |  |  |  |  |
| 1 | 59.38 (23.07) | 76.42 (11.83) | 66.78 (17.01) | 39.56 (18.27) | 41.01 (30.66) |
| 2 | 44.90 (31.04) | 86.99 (18.74) | 52.34 (25.05) | 13.41 (13.26) | 24.82 (25.61) |
| 3 | 36.97 (34.80) | 70.73 (23.79) | 35.67 (32.83) | 5.28 (16.11) | 69.50 (18.16) |

T: true; F: false; SD: standard deviation.
